# Accurate Finite Element Simulations of Dynamic Behaviour: Constitutive Models and Analysis with Deep Learning

**DOI:** 10.3390/ma17030643

**Published:** 2024-01-28

**Authors:** Yiwei Zhang, Chengcheng Guo, Yahui Huang, Ruizhi Zhang, Jian Zhang, Guoqiang Luo, Qiang Shen

**Affiliations:** 1 Hainan Institute, Wuhan University of Technology, Sanya 572000, China; 2 State Key Laboratory of Advanced Technology for Materials Synthesis and Processing, Wuhan University of Technology, Wuhan 430070, China

**Keywords:** constitutive model, FE simulations, deep learning, dynamic behaviour, Hugoniot elastic limit

## Abstract

Owing to the challenge of capturing the dynamic behaviour of metal experimentally, high-precision numerical simulations have become essential for analysing dynamic characteristics. In this study, calculation accuracy was improved by analysing the impact of constitutive models using the finite element (FE) model, and the deep learning (DL) model was employed for result analysis. The results showed that FE simulations with these models effectively capture the elastic-plastic response, and the ZA model exhibits the highest accuracy, with a 26.0% accuracy improvement compared with other models at 502 m/s for Hugoniot elastic limit (HEL) stress. The different constitutive models offer diverse descriptions of stress during the elastic-plastic response because of temperature effects. Concurrently, the parameters related to the yield strength at quasi-static influence the propagation speed of elastic waves. Calculation show that the yield strength at quasi-static of 6061 Al adheres to y = ax + b for HEL stress. The R-squared (R^2^) and mean absolute error (MAE) values of the DL model for HEL stress predictions are 0.998 and 0.0062, respectively. This research provides a reference for selecting constitutive models for simulation under the same conditions.

## 1. Introduction

Understanding the dynamic behaviour of metals is crucial for engineering applications, which are widely employed in defense [1], construction [2,3], machinery [4] and aerospace [5]. Studying the elastic–plastic response is vital for analysing palling, facilitating alloy design and supporting various other applications [6,7,8]. Importantly, investigating the dynamic response under uniaxial strain loading conditions provides valuable insights into metal behaviour, enhancing our ability to establish key parameters for engineering applications [9]. In the gas gun or split-Hopkinson pressure bar (SHPB), this response emerges when uniaxial strain loading surpasses the HEL threshold, resulting in the transforming of a single shock wave into faster elastic and plastic waves, ultimately pushing the material into an elastic–plastic state [10]. Meyers initially established a link between HEL stress and the dynamic yield strength as well as the Poisson’s ratio of materials [11]. Subsequent research by many investigators delved into the study of HEL in metals [12], revealing its strong dependence on factors, such as sample size [13], composition [14,15], impact mode [16], temperature [17], and strain rate [18]. Understanding the elastic–plastic behaviour is crucial for comprehensively understanding the dynamic response of materials under extreme conditions.

FE simulations are typically employed by researchers to investigate the dynamic behaviour of metals that are challenging to obtain through experiments [19,20,21]. Establishing an accurate FE model is paramount to ensure the precision of simulation [22,23,24]. The FE model in the dynamic behaviour of metal often improves the calculation accuracy by optimizing the algorithm [25,26] and, fine model mesh [27,28] and improving the reduction degree of the simulation model [29]. Piotr et al. investigated the capabilities and limitations of five different numerical approaches to improve the accuracy of modeling large strains of mushrooming projectiles and suggested FE for simulating projectile impact because of the CPU requirements [26]. Hakim et al. used FE simulation to explore the behaviour of impact and compression after the impact of tapered composite laminates and noted that compression after impact strength is underpredicted, which depends on the quality of meshing in the model of the transition region [27].

The methods mentioned above offer technical solutions to enhance accuracy, while some researchers also improve simulation accuracy through analysis of the strength of the material [30,31,32,33,34,35,36]. In the simulation, this strength is described by the constitutive model. Xiao observed that when assessing the dynamic behaviour of glass mat or fiber-reinforced polypropylene composites, the composite damage model (CODAM)constitutive model outperforms the use of compression and shear damage parameters [37]. In the context of dynamic processes causing ductile fracture in polycrystalline solids, Eftis et al. have developed a constitutive microdamage model to reasonably describe the high shock compression and the subsequent material microdamage and fracture [38]. Certain studies involve comparisons of multiple constitutive models to identify the most appropriate model for enhancing calculation accuracy. Doney et al. conducted a comprehensive study on the influence of various constitutive and state models on copper-shaped charge jets. The findings indicate that different constitutive models can impact the temperature and morphology of the jet [30]. Bobbili et al. demonstrated that these models offer distinct descriptions under different strain rates, with the JC model providing the most accurate flow stress prediction at 830 m/s [39]. Signetti et al. conducted a comprehensive review of constitutive models employed in the transition regime between high-velocity and hypervelocity impact in metals over the last seventy years. They identified the Johnson-Cook (JC) [40], Zerilli-Armstrong (ZA) [41], and Cowper-Symonds (CS) [42] models as particularly exemplary. It is worth noting that different constitutive models offer distinct descriptions of the elastic-plastic response in numerical simulations [43].

However, their results are phenomenological and do not explain for the varying constitutive descriptions of the dynamic behaviour of metal. Researchers have employed constitutive model parameters in various fields to elucidate diverse phenomena [44,45]. Similarly, this approach can be applied to simulations of the dynamic behaviour of metals.

In this paper, the elastic-plastic response of metals is the research focus, and a flyer plate impact model with CS, JC, and ZA models is developed using Autodyn-2D software 19.0 [46,47] to study the influence of the constitutive models and model parameters on the FE simulation. Data-driven numerical simulations can predict complex problems, providing resources and time-saving [48,49,50]. Here, with the establishment of a DL model, we thoroughly investigate the impact of constitutive models and their parameters, derived from extensive FE calculations, on the simulation results. This approach elevates the scientific rigor of our conclusions, emphasizing the thorough consideration of the impact of constitutive model parameters on the elastic-plastic response. It provides a quantitative analysis of their relationship, filling the existing gap in the comprehensive study on the elastic-plastic response of the material to various constitutive parameters.

## 2. Simulation Methods

### 2.1. Modeling

**Figure 1:**
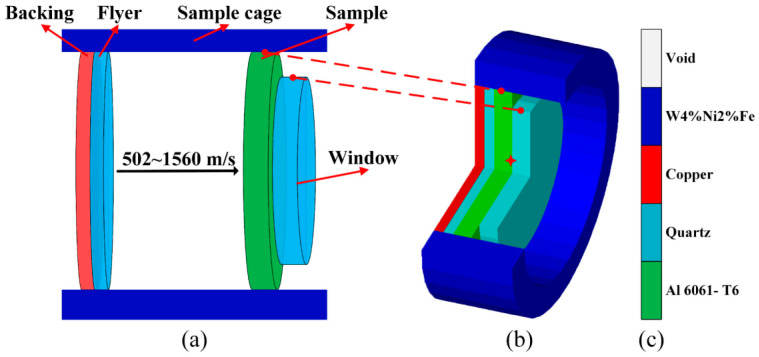
Constructions of the simulation model, including (**a**) schematic diagram of flyer plate impact, (**b**) flyer plate impact model constructed in Autodyn (the red star is the gauge), and (**c**) the material legend.

It was observed that the elastic-plastic response and HEL point in 6061 Al for shock compression over the stress range of 4–22 GPa were effectively captured by Huang and Asay’s quasi-elastic recompression experiments [51]. Recompression experiments are a common method to test the dynamic strength of materials, and they provide a means to observe the complex elastic–plastic behaviour of materials. The experimental setup and schematic diagram of the experimental device, along with the simulation model for flyer plate impact, are illustrated in Figure 1. The Cu and 6061 Al were chosen as backing and flyer materials and were used to induce recompression from the shocked state. Quartz has an elastic limit of about 6 GPa, making both the initial stress loading of 4 GPa and subsequent recompression to about 6 GPa elastic, simplifying the analysis of reloading. Therefore, quartz was chosen as the window material. In Autodyn, a two-dimensional axisymmetric model was constructed to replicate flyer plate impact within a gas gun. To precisely replicate the experimental configuration, the Euler void part was introduced to accommodate the vacuum conditions within the gas gun’s target chamber. The gun barrel material was represented by the Lagrange part, while other components were simulated using the Euler void part. Flyer plate impact simulations were carried out at 502, 660, 1150, and 1560 m/s. The entire loading process was computed using the Euler algorithm, and gauges were strategically positioned at the rear interface of the 6061 Al sample. To ensure precise computational results, the number of Euler part was meticulously set to 1,152,000.

### 2.2. Constitutive Model

The studied constitutive models include the JC, CS, and ZA models, which are widely employed for describing the elastic-plastic response of metals. According to Stefano’s introduction to the simulation of transition regime impact, these three models have demonstrated strong performance at elastic-plastic velocities [35].

The JC model is widely recognized for its comprehensive applicability as a phenomenological flow stress model, which takes into account temperature, strain and strain-rate [40]. The formula of the JC model is presented as follows:

(1) $$\sigma =\left(A+B\varepsilon^{n}\right)\left(1+Cln{\dot{\varepsilon }}^{*}\right)\left(1-T^{*m}\right)$$

Here, *σ* represents the equivalent flow stress, while *ε* stands for the equivalent strain. The parameters *A*, *B*, *C*, *m*, and *n* carry specific significance within the context of the model. *A* corresponds to the quasi-static yield stress at, *B* signifies the coefficient of strain hardening, *n* represents the strain hardening exponent, and *C* along with *m* are material constants denoting the coefficient of strain-rate hardening and thermal softening exponent, respectively. The parameters for 6061 Al used in the constitutive model are provided in Table 1 [52].

A connection between the dynamic flow stress and strain-rate is established in the CS model using the concept of equivalent plastic strain. Temperature and strain-rate effects are not accounted for in the CS model [42]. The expression representing the CS model is as follows:

(2) σ=A+Bεn1+ε˙D1q

Here, *σ* represents the uniaxial flow stress, while (*A* + *Bε^n^*) denotes the quasi-static flow stress, and *D* and *q* are material constants involved in the model. The specific constants for 6061 Al are provided in Table 2 [53].

The ZA model is derived based on dislocation mechanisms, which significantly influence the elastic behaviour and flow stress of metals under varying load conditions. The ZA model considers the impact of strain hardening, strain-rate hardening and thermal softening on the flow characteristics of metals [41]. For BCC materials, as expressed in the following:

(3) $$\sigma =C_{0}+C_{1}exp\left(-C_{3}T+C_{4}T\;ln\dot{\varepsilon }\right)+C_{5}\varepsilon^{n}$$

and for FCC materials, it is expressed as follows:

(4) $$\sigma =C_{0}+C_{2}\varepsilon^{\frac{1}{2}}exp\left(-C_{3}T+C_{4}T\;ln\dot{\varepsilon }\right)$$

where *σ* represents the equivalent flow stress. $C_{0}$, $C_{1}$, $C_{2}$, $C_{3}$, $C_{4}$ and n are material constants. The parameters for 6061 Al used in the constitutive model are presented in Table 3 [54].

### 2.3. Back Propagation Neural Network

**Figure 2:**
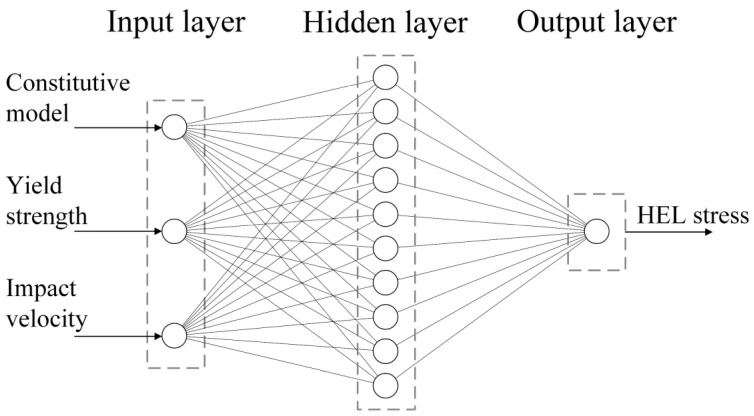
The HEL stress of 6061 Al prediction model based on ANN.

The BP model comprises an input layer, hidden layer, and output layer. The input layer is responsible for acquiring variables, and the hidden layer activates the data from the output layer before transmitting it to the output layer [55,56]. In this study, the types of constitutive models, velocity of impact, and quasi-static yield strength are taken as input variables, with the output being the HEL stress. Thus, the ANN has 3 neurons in the input layer and 1 neuron in the output layer, as illustrated in Figure 2, representing the overall structure of the model.

## 3. Results and Discussion

### 3.1. Effects of Constitutive Models and Model Parameters

A comprehensive comparison was conducted between numerical simulation results using JC, CS, ZA, and EP (elastoplastic) constitutive models and the experimental data provided by Huang and Asay [51] at 502 m/s and 1560 m/s to validate the accuracy and reliability of our model against their experimental results.

**Figure 3:**
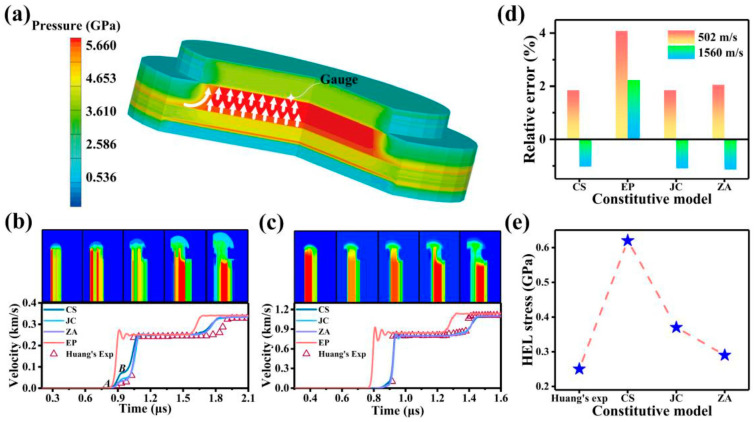
Particle velocity curve and contour of the flyer plate impact simulation results. (**a**) 3D pressure contour, white arrows indicate the direction of impact. Here (**b**,**c**) depict the analysis involving a comparison between the simulation results and Huang and Asay’s experimental profiles of particle velocity and pressure contours on the rear interface of the 6061 Al sample at 502 m/s and 1560 m/s. (**d**,**e**) present the relative error of maximum velocity with three models, along with the HEL stress obtained from different models at 502 m/s.

Figure 3b–d illustrates the maximum final velocities for 6061 Al at the rear interface, as determined by the JC, CS, ZA, and EP models at velocities of 502 m/s and 1560 m/s. The obtained results reveal that the relative errors in final velocities from the JC, CS, and ZA models consistently remain below 3% when compared with Huang and Asay’s data, highlighting the efficacy of the simulation model. The comparison of particle velocity curves clearly demonstrates a significant enhancement in the accuracy of simulating the elastic-plastic response when employing the JC, CS, and ZA models compared with the EP model.

Upon scrutinizing Figure 3b,c, a substantial distinction within the elastic-plastic response of the particle velocity profiles across the simulations using different constitutive models becomes evident. In Figure 3b, segment AB corresponds to the elastic precursor, with point B signifying the HEL. The HEL stress is represented as follows:

(5) $$\sigma_{HEL}=\rho_{0}C_{L}\mu_{p1}$$

(6) $$\mu_{p1}=\frac{1}{2}\mu_{fs}{|}_{B}$$

where *σ_HEL_* is the HEL stress, *μ_p_*_1_ is the particle velocity of elastic precursor AB and *μ_fs_* is the particle velocity of point *B* and *ρ*_0_ and *C_L_* are the density and longitudinal velocities of 6061 Al, respectively. Figure 3e displays the HEL stresses for the experimental and three models at 502 m/s. Through a comparison with the experiment, the ZA model emerges as the most appropriate choice for accurately describing the elastic-plastic response of 6061 Al given its error of 16.0%, with a 26.0% accuracy improved compared with other models.

**Figure 4:**
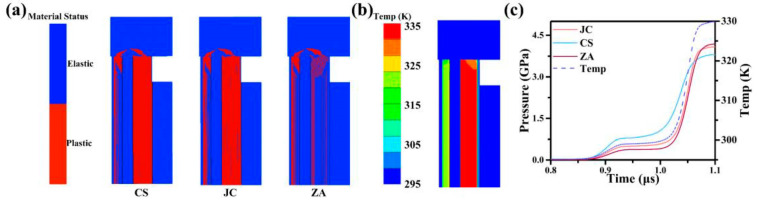
Status of 6061 Al at HEL. (**a**) The elastic-plastic distribution of 6061 Al at HEL with the CS, JC, and ZA models separately. (**b**) The temperature distribution of 6061 Al at HEL. (**c**) The temperature and the pressure of 6061 Al during impact with the three constitutive models separately.

At 502 m/s, the HEL moment exhibits a distinct behaviour for 6061 Al in simulation with the CS model, where it transitions entirely to a plastic status in Figure 4a. This stands in contrast to the coexistence of elastic and plastic observed with ZA and JC models. Figure 4b demonstrates that the pressure obtained with the CS model is higher than that with the JC and ZA models, and during this process, the temperature of 6061 Al rises by about 40.0 K. This may be attributed to the omission of temperature-induced stress weakening in the CS model. The 6061 Al experiences a temperature rise, leading to an overestimation of stress in the simulation results of the CS model during the impact. This can explain why, during the elastic-plastic response, the use of the CS model makes it easier for 6061 Al to plastic. Furthermore, it is observed that the lateral of the 6061 Al sample is more predisposed to entering the plastic compared with the central region. This phenomenon may be attributed to the influence of the lateral rarefaction wave during the impact [57].

The elastic-plastic response of 6061 Al is clearly distinguishable with the three models. However, their individual accuracy varies. To uncover the reasons behind any disparities, the influence of each parameter within the constitutive model on elastic-plastic response was systematically investigated by adjusting these parameters within the same simulation framework. This involved varying them at 60%, 80%, 120%, and 140% of their initial values, resulting in a comprehensive set of 60 simulation tasks.

**Figure 5:**
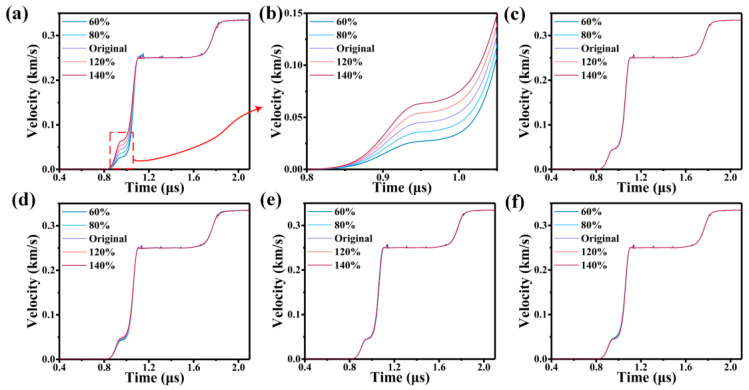
The impact of parameters of the JC model on particle velocity profiles at 502 m/s. (**a**) The impact of parameter A on particle velocity profiles at 502 m/s. (**b**) The impact of parameter A on particle velocity profiles at 502 m/s at the elastic-plastic response. (**c**–**f**) The impact of parameters B, C, m, and n on particle velocity profiles at 502 m/s.

In Figure 5, taking the JC model as an example, it is observed that only parameter A, representing the at quasi-static yield strength, significantly impacts the elastic-plastic response of the 6061 Al at 502 m/s. This relationship also holds true when analysing the CS and ZA models at 660, 1150, and 1560 m/s. Interestingly, none of the other parameters within the constitutive models exhibited any influence on the elastic-plastic response of 6061 Al during impact.

**Figure 6:**
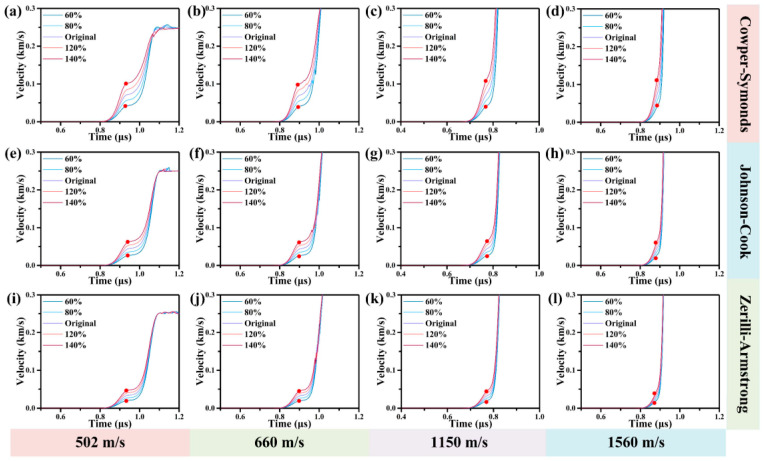
Correlation between the yield strength at quasi-static in JC, CS, and ZA models and the HEL Stress at 502, 660, 1150 and 1560 m/s. (**a**–**d**) depict the influence of quasi-static yield strength on HEL stress in the CS model at 502, 660, 1150, and 1560 m/s. (**e**–**h**) depict the influence of quasi-static yield strength on HEL stress in the JC model at 502, 660, 1150, and 1560 m/s. (**i**–**l**) depict the influence of quasi-static yield strength on HEL stress in the ZA model at of 502, 660, 1150, and 1560 m/s.

Figure 6 illustrates the influence of with the JC, CS, and ZA models on the particle velocity profiles of 6061 Al at 502, 660, 1150 and 1560 m/s and the red point represents the HEL stress. It is evident that HEL stress increases with the rise in the yield strength at quasi-static, although the three models exhibit different growth trends.

**Figure 7:**
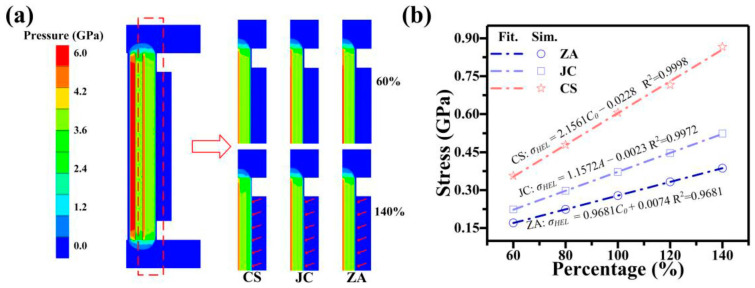
(**a**) Pressure contour of the HEL for 6061 Al after modifying the parameters of yield strength in CS, JC, and ZA at 502 m/s. (**b**) Yield strength at quasi-static and HEL Stress relationship of 6061 Al at 502 m/s.

In Figure 7a, the pressure contour reveals that an increase in the yield strength at quasi-static among different models leads to an early rise in pressure at the rear interface of the 6061 Al (highlighted by the red arrows). This phenomenon can be attributed to the heightened yield strength at quasi-static parameters intensifying the propagation of elastic waves during impact. This effect is most pronounced in the results obtained with the CS constitutive model, while it is weakest in the ZA model. Furthermore, the analysis of the constitutive model indicates that the change of yield strength at quasi-static conditions in the ZA model do not significantly affect other parameters, whereas alterations in the coefficients in the JC model and CS model impact the expression of strain-rate and temperature (terms with coefficients greater than 1) in Equations (1) and (2). This may explain why modifying ZA model parameters has the least impact on pressure contour.

As shown in Figure 7b, the data were further processed. A direct proportionality between the yield strength at quasi-static with three models and the HEL stress of the 6061 Al is revealed by the analysis. Interestingly, the ZA model calculates a significantly lower HEL stress compared with the other two models. This is consistent with the results of the analysis in the previous paragraph.

### 3.2. Analysis with the DL Model

The dataset of the DL model comprises 111 entries, divided into 84 for learning and 27 for prediction. Given the interrelation between HEL stress in the 6061 Al at 502, 554, 606 and 660 m/s, the type of constitutive model, yield strength at quasi-static, and impact velocity, these parameters have been selected as features for the dataset. The part of dataset is described in Table 4.

The BP model, as a self-supervised learning method, necessitates training with pre-acquired sample data to fit objective patterns. The number of hidden layer neurons crucially governs fitting: an insufficient count induces underfitting, while an excessive count leads to overfitting. Using regularization methods is essential to mitigate overfitting. In the context of flyer plate impact, where the functional relationship remains unknown, a rigorous exploration is undertaken for the selection of neurons in the hidden layer of the model.

In the selection of the number of neurons in the hidden layer, the most widely used approach is to determine its range based on empirical formulas as follows:

(7) $$l=\sqrt{n+m}+b$$

where *n* is the number of neurons in the input layer, *m* is the number of neurons in the output layer, and *b* is a constant between 1–10. Since the complexity of the prediction model for the HEL stress of 6061 Al during impact is fixed, the higher the compatibility with the BP model, the better the fitting effect of the model.

**Figure 8:**
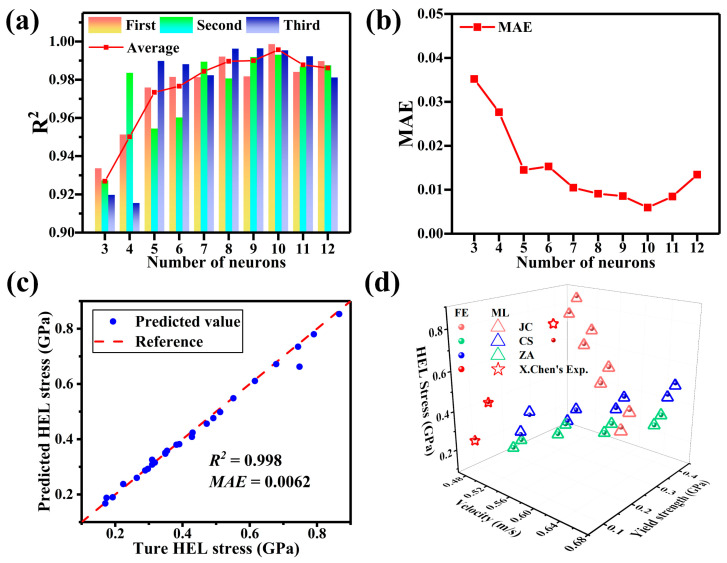
The influence of the numbers of neuron on the (**a**) coefficients for correlation and (**b**) MAE. (**c**) Plot of actual vs. predicted HEL stress for the testing set using the BP model, comparing FE and experimental datasets. (**d**) A total of 27 groups of predicted results for various.

From Figure 8a, it can be seen that the compatibility is optimal when the number of neurons is 10. Looking at the MAE in Figure 8b, it is observed that the average relative error in predicting the depth of the strengthening layer is minimized when the number of neurons is 10. Combining the R^2^ and MAE, it can be determined that when the number of hidden layer neurons is 10, the BP model achieves the optimal fitting effect for the prediction model of the HEL stress of 6061 Al.

To further confirm the accuracy of the model, a comparison was made between the HEL stress of aluminum obtained from 3 sets of X. Chen’s [58] experiments and 24 sets of untrained data generated through the FE calculations, with the model’s predicted results. The model exhibited strong predictive capabilities, as evidenced by the R^2^ value of 0.998 and MAE of 0.0062 for the prediction dataset, the predicted values also adhere to *y* = *ax* + *b*. The degree of fit is illustrated in Figure 8c,d.

It’s worth noting that, in the final fitting of the experimental data by X. Chen, all predictions were made using the ZA model. This decision was influenced by the significant errors observed when comparing the predicted values of the JC and CS models to the experimental data, which had a notable impact on the overall accuracy of the fitting. This confirms the conclusion from the previous chapter that the ZA model delivers the best performance in calculating HEL Stress. Compared with the FE analysis, the calculation time for the HEL stress prediction model trained using this DL has significantly reduced from 25 min to 43.6 s, with an average error of 1.046%.

## 4. Conclusions

In this study, the impact of the strength of materials on the FE simulation of metal impact is examined through the use of constitutive models and model parameters. The accuracy of the simulations is improved by establishing a reasonable and precise finite element model that incorporates these constitutive models. Furthermore, the FE and DL-driven analysis reveal the relationship between relevant physical quantities, and this approach can be applied to the discovery of other dynamic responses of metals, such as spalling and failure. The key findings are as follows:

1. Compared with the experiment, the ZA model exhibits superior accuracy in describing the elastic-plastic response of 6061 Al, and the accuracy of HEL stress with a 26.0% accuracy improved compared with other models at 502 m/s.
2. The quasi-static yield strength with the constitutive model critically governs the propagation intensity of elastic waves during the elastic-plastic response of 6061 Al. Notably, this influence is most conspicuous in the CS model and least pronounced in the ZA model, owing to each unique equation formulation.
3. The R^2^ and MAE of the BP model for predictions in the HEL stress of 6061 Al are 0.998 and 0.0062, respectively with an average error of 1.046%. This model effectively predicts HEL stress based on yield stress at quasi-static.
4. A linear relationship between yield stress at quasi-static and HEL stress during 6061 Al impact is unveiled through the combination of DL and FE analysis, expressed as *y* = *ax* + *b*. This relationship establishes a theoretical foundation for the development of related constitutive models.

## Figures and Tables

**Table 1:** Johnson-Cook model constants.

*A* (MPa)	*B* (MPa)	*N*	*C*	*m*
324	114	0.42	0.016	1.34

**Table 2:** Cowper-Symonds model constants.

*A* (MPa)	*B* (MPa)	*N*	*D*	*q*
291	451	0.66	15,202.02	19.73

**Table 3:** Zerillie-Armstrong model constants.

*C*_0_ (MPa)	*C*_2_ (MPa)	*C*_3_	*C*_4_	*n*
280	70.8	0.01	0.002	0.28

**Table 4:** The part of the dataset.

Constitutive Model	Yield Strength	Velocity	HEL Stress
JC (0)	0.2268	502	0.309774
CS (1)	0.3492	554	0.743622845
ZA (2)	0.28	606	0.288972109

(The types of constitutive models are denoted by 0, 1 and 2, respectively in the dataset.).

## Data Availability

The data presented in this study are available on request from the corresponding author.
